# Imatinib in gastrointestinal stromal tumour: Northern Cancer Network experience

**DOI:** 10.3332/ecancer.2010.162

**Published:** 2009-12-14

**Authors:** F Azribi, ARA Razak, P Dildey, J Adam, J Wilsdon, M Verrill

**Affiliations:** 1 Northern Institute for Cancer Research, Paul O’Gorman Building, University of Newcastle, Newcastle, NE2 4HH, UK; 2 Drug Development Programme, Suite 702, 7th Floor, Princess Margaret Hospital, 610 University Avenue, Toronto, ON M5G 2M9, Canada; 3 Royal Victoria Infirmary, Queen Victoria Road, Newcastle, NE1 4LP, UK; 4 Northern Centre for Cancer Care, Freeman Hospital, Freeman Road, Newcastle, NE7 7DN, UK

## Abstract

Imatinib treatment in metastatic or inoperable gastrointestinal stromal tumours (GIST) has shifted the paradigm of treatment of this disease. Successful clinical trials of imatinib led to rapid regulatory approval and, in England and Wales, National Institute for Health and Clinical Excellence (NICE) guidance on use of this technology. NICE recommend detailed audit of their guidelines in clinical practice. This audit reflects that guidance and was designed to document the use of imatinib in routine clinical practice.

**Methods:**

We conducted a retrospective audit of patients with GIST treated with imatinib from 1 February 2002 to 31 March 2007. Information gathered included patient demographics, disease characteristics and details of treatment administered, treatment response, toxicities and follow-up data. The primary objective was to record disease control rate (DCR), defined as a lack of progression on computed tomography at three months. Secondary end points of this audit were progression-free and overall survival. These were compared with published clinical trial results.

**Results:**

Thirty-six consecutive patients with a diagnosis of GIST treated with imatinib were identified. Median age of patients was 70.1 years. At the time of analysis, patients have been followed up for a median of 41.6 months. In total, patients were treated for a median of 15.8 months. Treatment was generally well tolerated with a small percentage of patients experiencing grade 3/4 toxicities. Disease control was observed in 30 patients (DCR, 83.3%, 95% CI 67.2–93.6, intention to treat analysis). The median progression free survival (PFS) in this cohort was 23.7 months (95% CI 12.9–34.4); while the median overall survival was 39.7 months (95% CI 22.8–56.5).

**Conclusion:**

Our data demonstrated that the treatment of unselected GIST patients within the NICE guidance compares favourably to previously published data of randomized registration studies of imatinib. Of note, the median age of this cohort is some ten years older than that reported in the trials. Imatinib was well tolerated with acceptable treatment-related adverse events.

## Introduction

The term gastrointestinal stromal tumours (GIST) was first coined by Mazur and Clark to describe gastrointestinal non-epithelial neoplasms that had neither smooth muscle nor Schwann-cell features [1]. They are thought to be derived from the interstitial cells of Cajal, which are the pacemaker cells of gastrointestinal tract [2].

Although GISTs are the commonest mesenchymal tumour of the gastrointestinal tract, they are relatively rare. Its exact incidence remains unknown, but is estimated at 14.5 cases per million in Sweden [3]. They commonly occur in people above the age of 50, with almost a similar distribution across the male and female gender [3,4]. GISTs are most commonly found in the stomach and small bowel, but they can occur in all other parts of the gastrointestinal tract as well as the abdominal and pelvic cavity. Liver and peritoneum are the most common sites of metastases [5]. The malignant potential of GIST depends on some features of the tumour, mainly size and mitotic rate [6]. The larger the size and the higher the mitotic rate, the more aggressive behaviour and risk of relapse the GIST will have. The location of GIST also has an impact on the outcome, with gastric tumours having a better prognosis than the intestinal tumours of the same features [7].

Definite diagnosis of this tumour requires histological confirmation. The landmark feature is the positive staining for CD117 receptor, also known as c-kit receptor. It is a transmembrane tyrosine kinase receptor for the cytokine stem cell factor. The majority of GISTs (circa 85%) expresses this protein. GISTs are often also positive for CD34, a cell surface protein also found in blood progenitor cells and endothelial cells. Less frequently, GIST cells are positive for smooth muscle actin (SMA), and they are rarely positive for the marker of cells derived from the neural crest (S100) [2,6,8,9].

Most GISTs have a gain of function mutation in the ***KIT*** proto-oncogene (***cKIT***) that translates into a constitutive ligand independent activation of the tyrosine kinase receptor [10]. This is thought to be the key event in the pathogenesis of GIST [11]. However, a minority of GISTs contain mutations in the homologous kinase platelet-derived growth factor receptor alpha (PDGFR-α) gene [12]. Mutations of these two closely related tyrosine kinases are mutually exclusive; with about 85–90% of GISTs, having a mutation in one of these two kinase genes [13]. The remaining GISTs carry the wild ***KIT*** gene type. To date, there are several mutations identified that have therapeutic implications. The most common ***cKIT*** mutation, exon 11, shows good response to the tyrosine kinase inhibitor imatinib and better overall survival (OS) than the less commonly occurring exon 9 mutation and the wild type GIST [14].

Surgery is the mainstay treatment for small-localized tumours. For locally advanced, inoperable or metastatic disease, tyrosine kinase inhibitor, imatinib mesylate, is the current worldwide standard first line treatment. It represents a revolution in the treatment of this notoriously chemotherapy-resistant tumour. Historically, the response rate to chemotherapy was very low, and the median survival for advanced disease was between 12 and 24 months [4,5,15]. Several phase 1 and 2 trials established the high efficacy and good tolerability of imatinib in treatment of GISTs [16–18]. A pivotal randomized trial conducted mainly in Europe showed an overall response rate of 52% and stable disease in 32% of patients [19]. The findings of the above trial was confirmed by another large trial, demonstrating an overall response rate of 45% while stable disease was achieved in 25% of patients [20]. With the use of imatinib, the median overall survival has improved dramatically, with a median overall survival exceeding 50 months [20,21]. The treatment protocol for patients in England and Wales with GIST is mandated by the National Institute for Health and Clinical Excellence (NICE) guidance (www.nice.org.uk/TA86/guidance). In essence, this guidance recommended: (i) treatment with imatinib for patients with unresectable/metastatic GIST at 400 mg daily under the supervision of cancer specialists with experience in the management of this disease; (ii) patients should be assessed on a 12-weekly basis and kept in treatment if the disease is responding to treatment, defined as stable disease or better; and (iv) patients who have progressed on imatinib therapy should have their treatment discontinued. NICE also recommended auditing the above guidance against local practices.

Despite the impressive results with imatinib treatment of this disease, little is known with regards to its efficacy in day-to-day clinical setting. We therefore undertook a retrospective audit of consecutive, unselected patients with GISTs who were treated with imatinib within the NICE guidance.

## Methods

### Patients

Potential patients were identified from the Northern Cancer Network (NCN) soft tissue and bone sarcoma multidisciplinary meeting (MDM) registry from 1 February 2002 to 31 March 2007. The NCN covers a population base of more than two million people and is responsible for oncological care for patients from the north-east of England. The NCN soft tissue and bone sarcoma MDM comprises dedicated pathologists, radiologists, surgeons and oncologists.

Patients with a histological confirmation of GIST who were treated with imatinib were included in this series. We examined patient demographics, disease characteristics and details of treatment administered. Information on treatment response, treatment failure, toxicities and follow-up were also collected. Each patient record was examined and data were retrieved for the above parameters. This audit was approved by the Caldicott Committee of the Newcastle upon Tyne Hospitals NHS Foundation Trust, which acts as the custodian of patient records and confidentiality at our institution.

### Treatment

Patients were treated as per recommendation of the NICE guidelines. All patients were treated with imatinib mesylate, 400 mg once daily p.o. unless specified otherwise. Patients were reviewed weekly for the first four weeks, then monthly for two-month and three-month visits from thereon. All patients had computed tomography (CT) evaluation pre-treatment, with three-month CT scans planned throughout treatment duration. More recently, a follow-up schedule with fewer visits has also been adopted.

### Audit end points

The primary end point of this retrospective audit was visual radiographic assessment for response and the clinical impression. This was evaluated as disease control rate (DCR), defined as a lack of clinically relevant progression on computed tomography (CT) at three months post-treatment initiation. We chose DCR as our primary end point as it reflects everyday clinical practice and it reflects accordance to the NICE guidelines. Whilst we also collated data on conventional response parameters (complete response (CR), partial response (PR), stable disease (SD) and disease progression (PD)), we acknowledge the limitations of response assessment, using conventional response parameters such as RECIST parameters in this disease [22].

Secondary end points of this audit included progression-free and overall survival. Progression-free survival (PFS) was defined as the time from start of imatinib treatment until documented progression or death from any cause. Overall survival was defined as the time from start of imatinib treatment to the time of the last review or death from any cause. Patients who were alive on the date of last follow-up were censored on both analyses.

### Safety profile

Common toxicity criteria according to the National Cancer Institute Grading System (version 3.0) were used in the evaluation of treatment toxicity.

### Statistical methods

The Kaplan-Meier method was used to generate estimation of progression-free and overall survival [23].

## Results

### Patient characteristics and treatment details

From 1 February 2002 to 31 March 2007, 36 patients with a diagnosis of GIST underwent treatment with imatinib within the Northern Cancer Network. Baseline characteristics of patients, their disease and previous treatment are summarized in Table 1. At the start of treatment with imatinib, 27 patients (75%) had metastatic disease, whilst five patients (13.9%) had locally advanced inoperable disease. Four patients (11.1%) were treated with imatinib despite having localized disease only as they were medically unfit for surgery.

In total, patients were treated for a median of 15.8 months. Two patients started imatinib at 200 mg daily p.o. as a precaution against bleeding problems. No patients had a dose of imatinib more than 400 mg o.d. Treatment deferral was documented in five patients due to toxicity. At the time of analysis, 26 out of 36 patients have had their imatinib treatment discontinued. Primary reason for treatment discontinuation was progressive disease (PD) (*n*=20), followed by treatment toxicity (*n*=6). All patients with progressive disease had their treatment discontinued.

### Treatment response and benefit

Thirty-four patients were evaluable for treatment response. Two patients (5.6%) were not evaluable for radiological response due to early clinical deterioration. DCR was observed in 30 patients (83.3 %, 95% CI 67.2–93.6, intention to treat analysis). There were no CR, whilst progressive disease was noted in four patients (11.1%). Treatment failure denoted by progressive disease on radiological assessment or the occurrence of early clinical deterioration occurred in six patients (16.7%).

**Figure 1:**
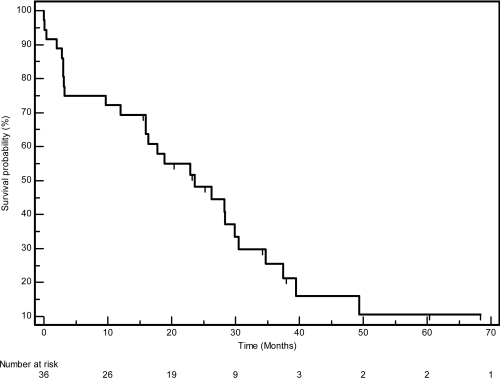
Progression-free survival curve—Kaplan-Meier plot for progression-free survival for all patients.

**Figure 2:**
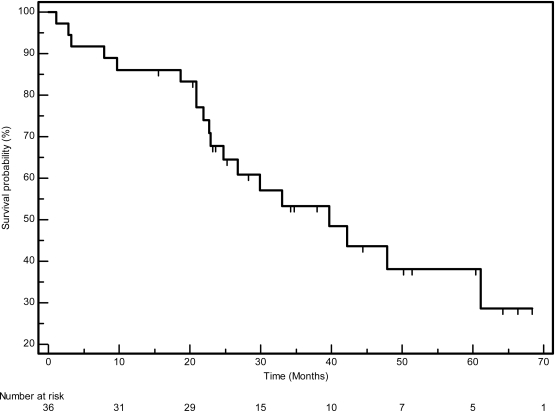
Overall survival curve—Kaplan-Meier plot for overall survival for all patients.

The median PFS in this cohort was 23.7 months (95% CI 12.9–34.4), whilst the median OS was 39.7 (95% CI 22.8–56.5). The PFS and OS actuarial plot is represented by Figures 1 and 2, respectively.

### Toxicity

In general, treatment with imatinib was tolerated well. There were no grade 3/4 haematological toxicities necessitating treatment deferral in our series. In 14 patients (38.8%), treatment was tolerated with no clinical toxicity. Non-haematological toxicities, when they occurred, were usually mild, with periorbital oedema (*n*=9), nausea (*n*=4), diarrhoea (*n*=3) and rash (*n*=3) being the commonest complain. However, a small proportion of patients developed grade 3/4 non-haematological toxicities and these are documented in Table 2.

## Discussion

The use of imatinib in the management of inoperable and metastatic GIST represented a sea change in the management of these tumours. For many years, GISTs were not recognized as a separate clinical entity from leiomyosarcomas [24], and it was only really when the opportunity for targeted therapy using imatinib in this disease was mooted that clinicians began to treat them as a separate clinical identity from other retroperitoneal sarcomas.

Following on from the success of imatinib in the management of chronic myeloid leukaemia [25], the role of this drug in GISTs was then established with a series of rapidly accruing phase 1, 2 and 3 trials conducted in parallel in Europe and North America, leading to the rapid licensing of the drug followed by approval in the United Kingdom from NICE.

One of the inevitable consequences of this rapid regulatory approval was that clinicians treating the disease knew the sometimes dramatic effect of imatinib treatment, but very few had experience of using the drug in real life. One distinct clinical group, the extreme elderly, who many would have not considered clinical trial material and their treatment of GIST patients on imatinib is response assessment. As alluded to earlier, we have chosen DCR as the primary end point for response evaluation to imatinib as opposed to traditional end points such as RECIST as it may be inaccurate in this setting. However, at present, the use of positron emission tomography imaging is fast becoming the standard modality for response evaluation for this disease in many institutions, negating the shortcomings of CT response assessment [26,27].

The NCN soft tissue and bone sarcoma group took an early lead in making imatinib available for patients in the region and the contribution of sarcoma, gastrointestinal (both upper and lower) and hepatobiliary surgeons to regular meetings along with the presence of specialist sarcoma and gastrointestinal pathologists, radiologists and oncologists undoubtedly contributed to the ability to manage these patients effectively.

This series represents the results of multi-disciplinary management of this group of patients. The management guidelines were, essentially, fixed by the NICE guidance on the management of GIST, which is applicable locally and has been followed closely. This has enabled the generation of detailed individual patient data on response and treatment outcome. Our response rate (to include patients who had partial response and stable disease), time to progression and overall survival map almost exactly onto the published trial data from both the EORTC and the American Intergroup studies and show that in a multi-disciplinary setting in a UK centre, it is possible to match the results of clinical trials in the population as a whole, not just in those patients who would be eligible for clinical trials. Our results underpin both the efficacy and safety of imatinib treatment despite the high median age of patients in the cohort. This age distribution makes our experience unique among the published series.

Our unexpected finding of cardiac toxicity in two of the patients in the series highlights the importance of continuing pharmaco-vigilance after product licensing. This was not a finding that was expected from the registration studies and may, in part, be a reflection of the age group treated. It raises the question of a need to monitor cardiac function in selected patients although our review does not have the power to answer this.

## Conclusion

Our data show that in routine clinical practice based on a comprehensive multi-disciplinary team framework, the impressive results produced by imatinib in GISTs in the randomized pivotal trials can be reproduced. The treatment can be delivered safely and the response rates and other clinical outcomes map onto the published clinical trial data. This audit meets the NICE requirement for practice evaluation and our results confirm the appropriateness of the NICE guidance to UK practice.

## Figures and Tables

**Table 1:**
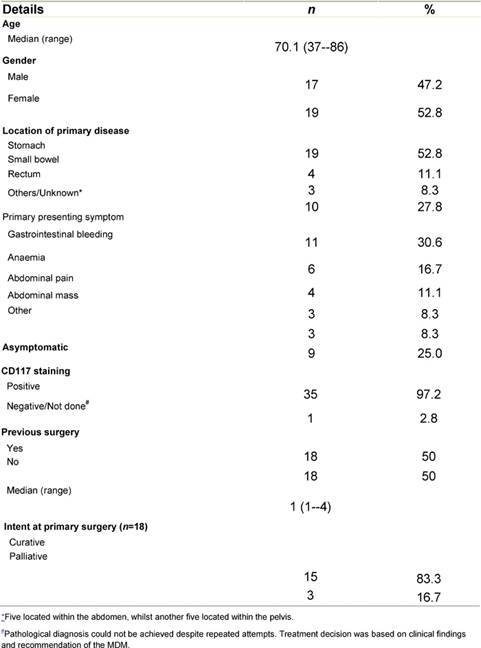
Patient, disease and previous treatment demographics characteristics of patients, disease and previous treatment

**Table 2:**
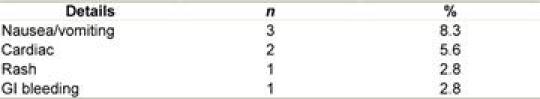
Grade 3/4 non-haematological toxicities
